# Survival by First-line Treatment Type and Timing of Progression Among Follicular Lymphoma Patients: A National Population-based Study in Sweden

**DOI:** 10.1097/HS9.0000000000000838

**Published:** 2023-02-23

**Authors:** Caroline E. Weibull, Tove Wästerlid, Björn Engelbrekt Wahlin, Per-Ola Andersson, Sara Ekberg, Sandra Lockmer, Gunilla Enblad, Michael J. Crowther, Eva Kimby, Karin E. Smedby

**Affiliations:** 1 Clinical Epidemiology Division, Department of Medicine Solna, Karolinska Institutet, Stockholm, Sweden; 2 Department of Hematology, Karolinska University Hospital, Stockholm, Sweden; 3 Unit of Hematology, Department of Medicine Huddinge, Karolinska Institutet, Stockholm, Sweden; 4 Section for Hematology and Coagulation, Sahlgrenska University Hospital, Gothenburg, Sweden; 5 Department of Immunology, Genetics and Pathology, Experimental and Clinical Oncology, Uppsala University, Uppsala, Sweden; 6 Department of Medical Epidemiology and Biostatistics, Karolinska Institutet, Stockholm, Sweden; 7 Red Door Analytics, Stockholm, Sweden

## Abstract

In follicular lymphoma (FL), progression of disease ≤24 months (POD24) has emerged as an important prognostic marker for overall survival (OS). We aimed to investigate survival more broadly by timing of progression and treatment in a national population-based setting. We identified 948 stage II-IV indolent FL patients in the Swedish Lymphoma Register diagnosed 2007–2014 who received first-line systemic therapy, followed through 2020. Hazard ratios (HRs) with 95% confidence intervals (CIs) were estimated by first POD at any time during follow-up using Cox regression. OS was predicted by POD using an illness-death model. During a median follow-up of 6.1 years (IQR: 3.5–8.4), 414 patients experienced POD (44%), of which 270 (65%) occurred ≤24 months. POD was represented by a transformation in 15% of cases. Compared to progression-free patients, POD increased all-cause mortality across treatments, but less so among patients treated with rituximab(R)-single (HR = 4.54, 95% CI: 2.76-7.47) than R-chemotherapy (HR = 8.17, 95% CI: 6.09-10.94). The effect of POD was similar following R-CHOP (HR = 8.97, 95% CI: 6.14-13.10) and BR (HR = 10.29, 95% CI: 5.60-18.91). The negative impact of POD on survival remained for progressions up to 5 years after R-chemotherapy, but was restricted to 2 years after R-single. After R-chemotherapy, the 5-year OS conditional on POD occurring at 12, 24, and 60 months was 34%, 46%, and 57% respectively, versus 78%, 82%, and 83% if progression-free. To conclude, POD before but also beyond 24 months is associated with worse survival, illustrating the need for individualized management for optimal care of FL patients.

## INTRODUCTION

Follicular lymphoma (FL) is the most common indolent lymphoma type, accounting for about 20% of all lymphomas.^1^ FL is predominantly incurable, with the exception of limited-stage disease which may be cured with radiotherapy (RT).^2^ In recent years, the heterogeneity of FL has become increasingly recognized, both regarding tumor molecular characteristics and clinical course. Due to the incurability of advanced-stage FL even with modern therapy, and the fact that early therapy has not been shown to prolong survival, many patients with indolent FL are managed with an initial watch-and-wait approach.^1,3,4^ However, some patients require immediate systemic treatment due to rapidly progressing or symptomatic disease. During recent decades, both progression-free and overall survival (PFS/OS) have improved following the introduction of monoclonal anti-CD20 antibody therapy (mainly rituximab, R), and improved supportive care.^5–7^ Importantly, these results have also translated into improvements in lymphoma-specific survival outside the clinical trial setting, as illustrated in 2 European population-based studies.^8,9^ Treatments of choice include R as a single agent or in combination with chemotherapy (eg, with bendamustine, BR) or in cases where a fast tumor reduction is needed, R-CHOP (cyclophosphamide, doxorubicin, vincristine, and prednisone).^1,3^ Still, some patients do not respond to therapy and suffer from rapidly progressive disease and risk of transformation to diffuse large B-cell lymphoma.^10^

Factors that determine early disease progression and need for treatment are not yet entirely defined. Currently, patients with FL are risk stratified using FLIPI, FLIPI2, and m7-FLIPI, which take into account clinical and laboratory characteristics and molecular features in the latter,^11–13^ but these are still not sufficient to predict need for treatment and risk of progression, why novel scoring methods are being developed.^14^ Several studies have found that FL patients in need of initial systemic treatment and subsequent early re-treatment within 2 years of diagnosis (POD24) have a particularly bad prognosis.^15–20^ The impact of POD24 on prognosis was initially established for patients treated with R-chemotherapy,^15,18,21,22^ but has since been demonstrated to predict outcome also among patients managed with immunotherapy only.^16,23–25^ However, the majority of reports on the impact of POD24 have been performed on patients from clinical trials or from single centers, and comprehensive real-world evaluations of the impact of timing of progression are lacking. Also, the proportion of patients that experience POD24 appears to vary by treatment, as does the association of POD24 with risk for transformation.^17,24^ For example, lower rates of progression of disease (POD) have been reported for BR-treated patients, but with a concomitantly higher risk of transformation among BR-treated patients who do experience POD24.^18,26^ In contrast, the risk of experiencing POD24 appears to be higher among patients managed with immunotherapy only,^16,23,24^ but the impact on survival among these patients is less pronounced.^17^

The aim of the current study was to investigate the impact of timing of first progression on survival more broadly both before and beyond the POD24 time point, and by type of first-line systemic treatment in a large Swedish population-based cohort of FL patients diagnosed 2007–2014 and followed through 2020.

## MATERIAL AND METHODS

### Data sources and data collection

Patients registered with a first diagnosis of indolent FL between 2007 and 2014 were identified through the Swedish Lymphoma Register (SLR). Discordant and primary cutaneous FL patients were not included. The SLR was initiated in 2000 and has a coverage above 95% compared to the nationwide Swedish Cancer Register.^27^ The SLR records detailed data on patient and clinical characteristics at diagnosis, first-line treatment, and progression. For this study, the register data were validated and supplemented with data on progression, transformation, and second-line therapies from a medical chart review. Informed consent was enquired for all patients alive by October 18, 2018, except in 2 Swedish regions where the ethical permission allowed for complementary data collection without active consent (Skåne and Uppsala). For deceased patients, no informed consent was needed from next-of-kin. The medical chart data collection was initiated in 2018 and finalized in 2020.

### Study population

**Figure 1.**
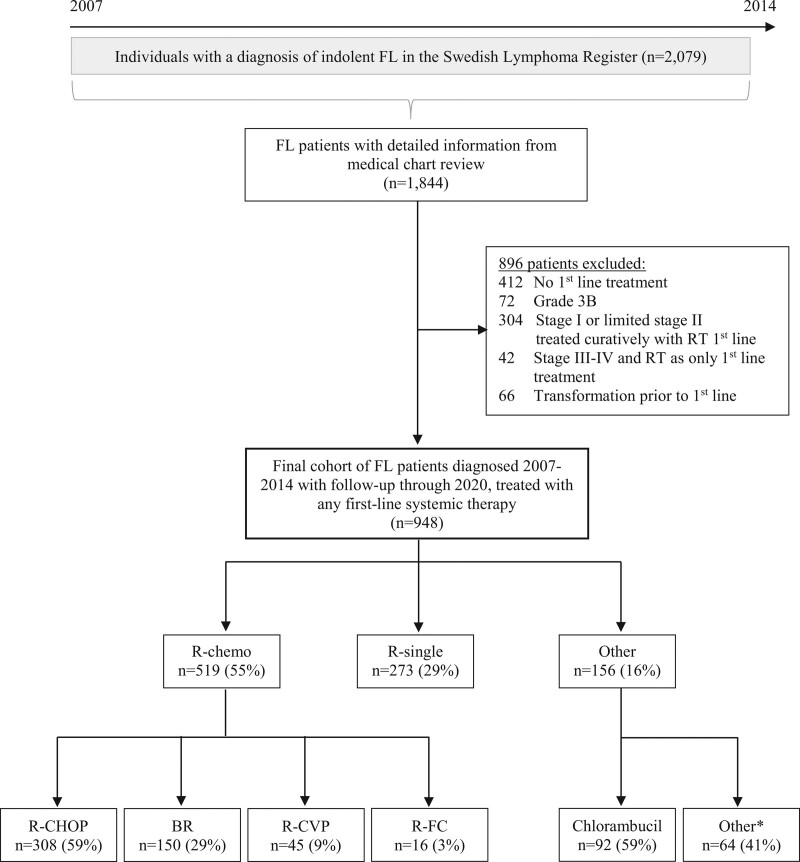
**Flowchart of included patients and reasons for exclusions and distribution of first-line treatment among patients diagnosed with indolent FL in Sweden 2007-2014.** *Among whom 27 patients (42%) received CHOP/B/CVP/FC without R and remaining 37 patients (58%) received oral chemotherapy such as trophosphamide, or lenalidomide, or had missing information on type of systemic treatment. B = bendamustine; chemo = chemotherapy; CHOP = cyclophosphamide, doxorubicin, vincristine, and prednisone; CVP = cyclophosphamide, vincristine, and prednisone; FC = fludarabine and cyclophosphamide; FL = follicular lymphoma; R = rituximab.

A total of 2079 registered patients were identified as eligible for the study and the medical chart review could be completed for 1844 FL patients (89%). Noncompletion was primarily due to a lack of active consent, missing medical records, and cases found to be discordant (transformed) at diagnosis although registered as indolent. Patients who were managed with watch-and-wait only during the entire follow-up period (n = 412), had grade 3B (n = 72), were stage I or limited stage II and treated with curative intent RT (n = 304), were stage III-IV disease and treated with RT only (n = 42), and patients who experienced a transformation before first-line treatment initiation (n = 66) were excluded. The final study population comprised 948 patients (Figure 1).

### Definition of treatment groups

Patients were classified as treated first-line with either R-chemotherapy (R-chemo), R-single, or other treatments. The R-chemo group included R-CHOP, BR, R-CVP (cyclophosphamide, vincristine, prednisone), and R-FC (fludarabine, cyclophosphamide). Standard dose of bendamustine in Sweden during the study period was 90 mg/m^2^. The “other” group consisted of patients treated with chemotherapy (CHOP/B/CVP/FC) without R, oral treatments such as chlorambucil, trophosphamide or lenalidomide, and patients with missing information on type of systemic treatment.

### Definition of progression of disease

POD was defined as either progressive disease as best response to first-line therapy, initial response but later relapse/progression as indication for second-line therapy, or transformation. Patients not (yet) fulfilling any of these criteria were considered progression-free (PF). Date of POD was subsequently defined as date of first-line evaluation, second-line treatment initiation date, or date of first transformation (after first-line treatment). POD24 was defined as fulfilling any of the above criteria within 24 months after start of first-line treatment.

### Statistical methods

Frequencies and proportions of demographical, clinical, and follow-up characteristics were calculated by type of first-line treatment. Chi-square tests were used to test difference in distribution between categorical variables. Among patients with POD, the proportion of patients where POD (and POD24) was represented by a transformation was calculated by first-line treatment. Follow-up ended at date of death due to any cause, or end of study period, whichever came first. End of study period depended on when the data collection was performed, and varied between December 31, 2018, and December 31, 2020. Follow-up was restricted to the first 10 years following diagnosis. The POD rate was predicted by first-line treatment type from a univariable flexible parametric survival model allowing for non-proportional effects, with 4 degrees of freedom for both the baseline rate and the time-dependent effect.

The prognostic impact of POD was analyzed in 2 ways. To estimate the relative effect of POD on all-cause mortality by first-line treatment, Cox regression models were fitted treating POD as a time-varying exposure. Both univariable (adjusted for time since first-line treatment) and multivariable models (adjusted for age and calendar year of diagnosis, sex, and FLIPI) were fitted. Interaction effects between POD and treatments were tested using Wald tests. To further estimate the prognostic value of progression as a function of time of POD/PF since first-line treatment on the absolute risk scale, and hence, quantify the impact of timing of POD on survival, the 5-year OS conditional on either being PF or having experienced POD at different time points during follow-up was estimated. This was done using an illness-death model^28^ with three states: diagnosed and treated for FL and still PF and alive (initial state), progressed and alive (POD state), and deceased (DEAD state) (Suppl. Figure S1). Transition rates from the initial state to the POD or DEAD state, and from POD to DEAD, were modeled using flexible parametric models. Time since initiation of first-line treatment was used as the underlying time scale, and the transition from POD to DEAD was additionally adjusted for time of POD (ie, semi-Markov). We separately assessed POD24 since previous studies have focused on this time point. Follow-up started at date of progression among POD24 patients, and at 24 months after treatment initiation among patients who were PF as in previous studies.^21,29–31^ In the PF patient group, patients dying (n = 74) or being censored due to the end of study period (n = 6) within 24 months were excluded from this analysis (n = 80). OS was estimated using the Kaplan–Meier method by POD24 for the different treatment groups. Last, we compared the lymphoma-specific and other-cause mortality between progressed and nonprogressed patients, using similar models as for the all-cause mortality analyses.

## RESULTS

In the final cohort of 948 patients, 519 (55%) were treated with R-chemo, 273 (29%) with R-single, and 156 (16%) with other treatments (Figure 1; Table 1). The “other” treatment group consisted of patients treated with chlorambucil (n = 92/156, 59%), CHOP/B/CVP/FC without R (n = 27/156, 17%), or other oral chemotherapy agents/missing treatment information (n = 37/156, 24%). Among patients treated with R-CHOP, 263 (85%) received six or more treatment cycles, and 138 (45%) received maintenance treatment with anti CD20 antibody treatment (mainly R). A majority of BR patients (n = 137, 91%) were treated with four or more cycles, and 44 (29%) received R maintenance.

**Table 1:** Demographic and Clinical Characteristics of Patients With Indolent Follicular Lymphoma Treated With Any First-line Systemic Therapy, by Treatment Type

Variable	R-chemo^a^	R-CHOP	BR	R-single	Other^b^	Total
Overall	519 (100)	308 (100)	150 (100)	273 (100)	156 (100)	948 (100)
Year of diagnosis, n (%)						
2007–2010	252 (48.5)	189 (61.4)	17 (11.3)	127 (46.5)	92 (59.0)	471 (49.7)
2011–2014	267 (51.5)	119 (38.6)	133 (88.7)	146 (53.5)	64 (41.0)	477 (50.3)
Age at diagnosis, n (%)						
≤49	62 (12.0)	39 (12.7)	20 (13.3)	52 (19.1)	9 (5.8)	123 (13.0)
50–64	160 (30.8)	103 (33.4)	45 (30.0)	87 (31.9)	43 (27.6)	290 (30.6)
65–74	194 (37.4)	111 (36.0)	59 (39.3)	84 (30.8)	38 (24.4)	316 (33.3)
≥75	103 (19.9)	55 (17.9)	26 (17.3)	50 (18.3)	66 (42.3)	219 (23.1)
Median age (range)	66 (24-92)	65 (24-92)	66 (31-90)	64 (27-91)	71 (38-98)	66 (24-98)
Sex, n (%)						
Male	276 (53.2)	166 (53.9)	74 (49.3)	121 (44.3)	85 (54.5)	482 (50.8)
Female	243 (46.8)	142 (46.1)	76 (50.7)	152 (55.7)	71 (45.5)	466 (49.2)
Stage, n (%)						
Ann-Arbor II	97 (18.7)	57 (18.5)	32 (21.3)	57 (20.9)	33 (21.2)	187 (19.7)
Ann-Arbor III	177 (34.1)	103 (33.4)	51 (34.0)	108 (39.6)	41 (26.3)	326 (34.4)
Ann-Arbor IV	242 (46.6)	146 (47.4)	67 (44.7)	108 (39.6)	79 (50.6)	429 (45.3)
Missing	3 (0.6)	2 (0.7)	0 (0.0)	0 (0.0)	3 (1.9)	6 (0.6)
FLIPI, n (%)						
Low risk	86 (16.6)	44 (14.3)	33 (22.0)	68 (24.9)	31 (19.9)	185 (19.5)
Intermediate risk	132 (25.4)	77 (25.0)	41 (27.3)	105 (38.5)	57 (36.5)	294 (31.0)
High risk	298 (57.4)	187 (60.7)	74 (49.3)	96 (35.2)	61 (39.1)	455 (48.0)
Missing	3 (0.6)	0 (0.0)	2 (1.3)	4 (1.5)	7 (4.5)	14 (1.5)
Performance status, n (%)						
0	327 (63.0)	200 (64.9)	100 (66.7)	220 (80.6)	98 (62.8)	645 (68.0)
1	141 (27.2)	80 (26.0)	37 (24.7)	45 (16.5)	39 (25.0)	225 (23.7)
2+	38 (7.3)	19 (6.2)	11 (7.3)	5 (1.8)	17 (10.9)	60 (6.3)
Unclear	13 (2.5)	9 (2.9)	2 (1.3)	3 (1.1)	2 (1.3)	18 (1.9)
Grade, n (%)						
1	103 (19.9)	60 (19.5)	28 (18.7)	73 (26.7)	46 (29.5)	222 (23.4)
2	226 (43.6)	128 (41.6)	71 (47.3)	120 (44.0)	62 (39.7)	408 (43.0)
3A	111 (21.4)	78 (25.3)	26 (17.3)	51 (18.7)	22 (14.1)	184 (19.4)
Low-grade UNS	16 (3.1)	10 (3.3)	3 (2.0)	10 (3.7)	1 (0.6)	27 (2.9)
Unclear	63 (12.1)	32 (10.4)	22 (14.7)	19 (7.0)	25 (16.0)	107 (11.3)

a Patients treated with R-CHOP, BR, R-CVP, or R-FC.

b Patients treated with chlorambucil, other oral chemotherapeutic drugs, or CHOP/B/CVP/FC without rituximab.

B = bendamustine; CHOP = cyclophosphamide, doxorubicin, vincristine, and prednisone; CVP = cyclophosphamide, vincristine, and prednisone; FLIPI = Follicular Lymphoma International Prognostic Index; UNS = unspecified; R = rituximab.

The median time to primary treatment initiation was 47 days (interquartile range [IQR]: 22–133), and 205 (22%) patients started their treatment after 6 months or more. Median age at diagnosis was 66 (IQR: 58–73), 64 (55–71), and 71 (62–79) years for patients treated with R-chemo, R-single, and other treatments, respectively (Table 1). Stage distribution differed slightly across treatment groups, the proportion of stage IV for R-chemo, R-single, and other treatments were 47%, 40%, and 51%, respectively (*P* = 0.022). Patients treated with R-single had a lower proportion of high-risk FLIPI (35%) compared to patients treated with R-chemo (57%) and other treatments (39%) (*P*-value < 0.001). Increasing age, male sex, high-risk FLIPI, and poor performance status were associated with increased all-cause mortality (Suppl. Table S1).

**Figure 2.**
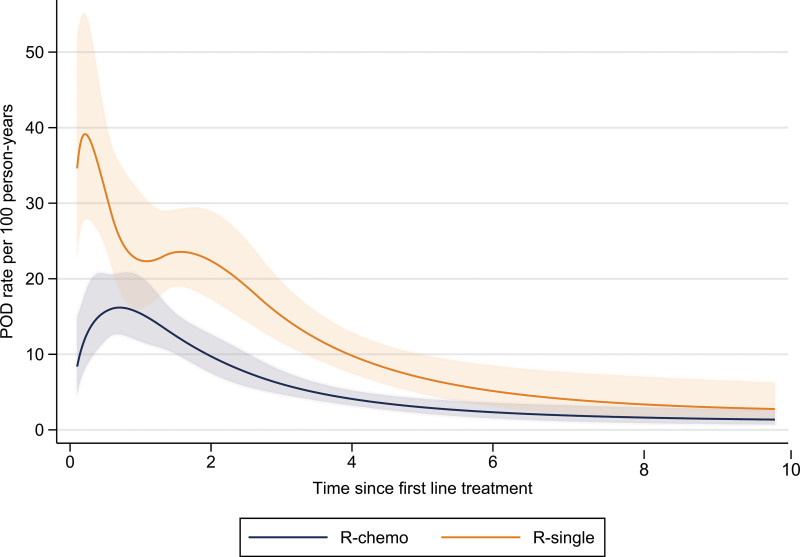
**Progression of disease (POD) rate per 100 person-years over time since first-line treatment, by first-line treatment type.** Predicted from univariable flexible parametric survival model with 4 degrees of freedom for both baseline rate and the time-dependant effect. Shaded areas represent the 95% CIs. CI = confidence interval; POD = progression of disease; R = rituximab.

The median follow-up time (from first-line treatment) was 6.1 years (IQR: 3.5–8.4), during which 414 (44%) patients experienced POD (Table 2). The proportion was higher in patients treated with R-single (60%) than with R-chemo (35%) (*P* < 0.001). Within the R-chemo group, 110 (36%) and 44 (29%) patients treated with R-CHOP and BR, respectively, experienced POD. The rate of POD was highest during the first 2 to 3 years following first-line treatment (Figure 2). Among all progressed patients, 270 (65% or 28% out of the total number of patients) occurred within 24 months of first-line treatment, with similar proportions for R-CHOP (61%) and BR (59%) but higher for R-single (68%). The proportion morphologically verified transformations at POD was 15%, and did not differ significantly by treatment (Table 2).

**Table 2:** Number and Proportions of Patients Experiencing POD During Follow-up After First-line Treatment, and Among These the Proportion of Progressions Within 24 Months of First-line Treatment (POD24), and the Number and Proportion of Morphologically Verified Transformation at POD and POD24

	POD			Transformation	
Treatment, n (row %)	Yes	Yes, within 24 mo (POD24)	No	Yes, at POD	Yes, at POD24
R-chemo^a^	179 (34.5)	110 of 179 (61.5)	340 (65.5)	32 of 179 (17.9)	21 of 110 (19.1)
R-CHOP	110 (35.7)	67 of 110 (60.9)	198 (64.3)	18 of 110 (16.4)	13 of 67 (19.4)
BR	44 (29.3)	26 of 44 (59.1)	106 (70.7)	9 of 44 (20.5)	5 of 26 (19.2)
R-single	165 (60.4)	113 of 165 (68.5)	108 (39.6)	19 of 165 (11.5)	13 of 113 (11.5)
Total	414^b^ (43.7)	270 of 414 (65.2)	534 (56.3)	62^c^ of 414 (15.0)	41 of 270 (15.2)

B = bendamustine; chemo = chemotherapy; CHOP = cyclophosphamide, doxorubicin, vincristine, and prednisone; col = column; R = rituximab; POD = progression of disease.

a Patients treated with R-CHOP, BR, R-CVP, or R-FC.

b All patients treated with any first-line immunochemotherapy.

c In addition to this number, 10 clinically suspected transformations occurred.

**Figure 3.**
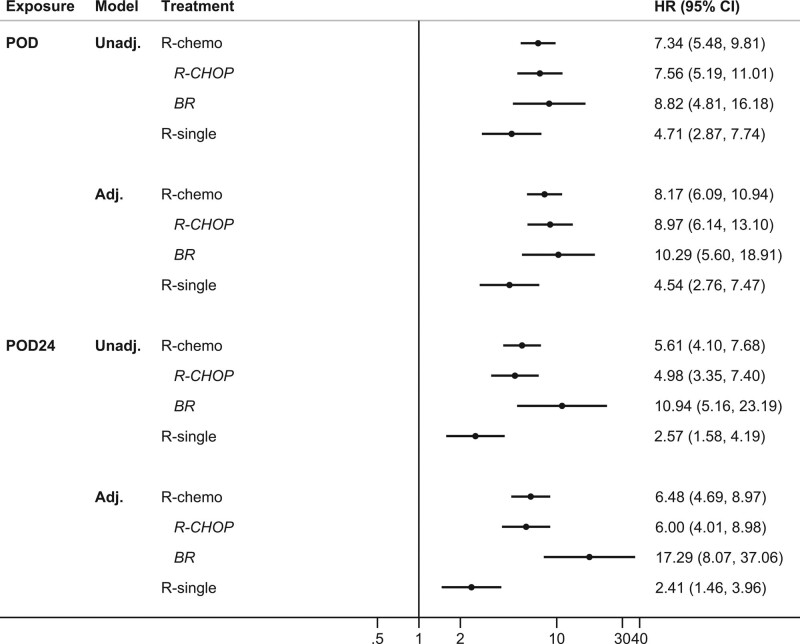
**Forest plot showing unadjusted and adjusted HRs with 95% CIs comparing all-cause mortality among patients with POD and patients still progression-free, by first-line treatment.** In the top panel, POD could occur at any time point during follow-up whereas in the bottom panel POD occurred within 24 months (POD24). Adj. = estimates from Cox proportional hazards models additionally adjusted for age at diagnosis (categorized), sex, calendar year of diagnosis (categorized), and FLIPI; B = bendamustine; CHOP = cyclophosphamide, doxorubicin, vincristine, and prednisone; CI = confidence interval; FLIPI = Follicular Lymphoma International Prognostic Index; HRs = hazard ratios; POD = progression of disease; R = rituximab; Unadj. = estimates from Cox proportional hazards models adjusted for time since first-line treatment.

Patients experiencing POD after R-chemo had an 8-fold increased all-cause mortality rate following POD compared to PF patients (HR_adj_ = 8.17, 95% CI: 6.09-10.94). For patients treated with R-single, the effect of POD was significantly smaller (*P* = 0.0420 from the Wald test of interaction, HR_adj_ = 4.54, 95% CI: 2.76-7.47) (Figure 3, top panel). Within the R-chemo treatment group, mortality was similarly increased among patients with POD after R-CHOP (HR_adj_ = 8.97, 95% CI: 6.14-13.1) as among patients treated with BR (HR_adj_ = 10.3, 95% CI: 5.60-18.9) (*P* = 0.7020). Similar results were seen in the analysis of POD24, but with a significantly stronger effect of POD24 in BR compared to R-CHOP treated patients: patients with POD24 after BR experienced a 17-fold increased mortality compared with PF patients, whereas among R-CHOP treated patients it was 6-fold increased (Figure 3, bottom panel, *P* = 0.0154). When investigating lymphoma-specific and other-cause mortality among progressed and nonprogressed patients, patients treated with R-chemo had a 42 times higher lymphoma-specific mortality rate compared with similar patients staying PF, and a 2.4 times higher mortality due to other causes (Suppl. Table S2). Patients who progressed following R-single had a highly increased rate of lymphoma-specific death, but not of other causes.

**Figure 4.**
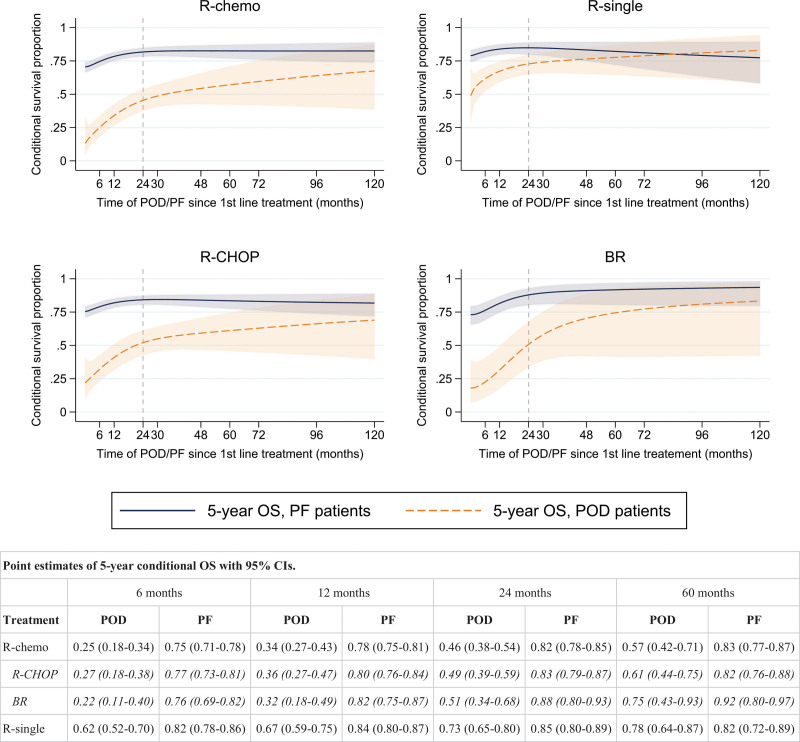
**Conditional 5-year survival among FL patients who experienced POD or were still PF, as a function of time point of POD/PF since first-line treatment initiation (in months), by first-line treatment type (top panel: R-chemo and R-single, bottom panel: R-CHOP and BR).** The vertical dashed line indicates POD24/PF24. The table shows point estimates of the 5-year conditional OS with 95% CIs for POD/PF at 6, 12, 24, and 60 months. B = bendamustine; chemo = chemotherapy; CHOP = cyclophosphamide, doxorubicin, vincristine, and prednisone; CI = confidence interval; CVP = cyclophosphamide, vincristine, and prednisone; FL = follicular lymphoma; OS = overall survival; PF = progression-free; POD = progression of disease; R = rituximab.

**Figure 5.**
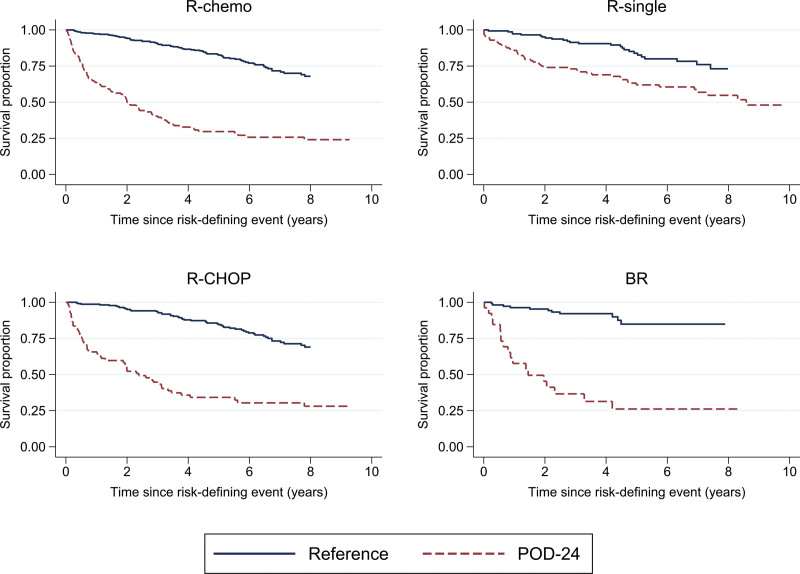
**OS estimated with the Kaplan–Meier method among patients with FL treated with R-chemo or R-single as first-line therapy, by POD24 (progression of disease within 24 months of first-line treatment initiation).** Follow-up started at date of progression for POD24-patients and at 24 months after treatment for progression-free patients (“reference”). Eighty patients not experiencing POD within 24 months were excluded due to death (n = 74) or administrative censoring (n = 6). Top panel: R-chemo and R-single, bottom panel: R-CHOP and BR. B = bendamustine; chemo = chemotherapy; CHOP = cyclophosphamide, doxorubicin, vincristine, and prednisone; FL = follicular lymphoma; OS = overall survival; POD = progression of disease; R = rituximab.

Figure 4 shows the 5-year conditional OS among progressed and PF patients by timing of POD/PF and treatment. A short time between first-line treatment and progression was associated with the lowest OS, irrespective of treatment type. For R-chemo overall, as well as R-CHOP and BR-treated patients, the negative impact of POD on OS remained for progressions occurring up to five years after first-line treatment. Among patients receiving any R-chemo who experienced POD at 12 months, the 5-year conditional OS was 34% (95% CI: 27%-43%), versus 78% (95% CI: 75%-81%) among PF. Among R-chemo-treated patients experiencing POD at 24 months and 60 months, the corresponding OS proportions were 46% (95% CI: 38%-54%) and 57% (95% CI: 42%-71%), compared with 82% (95% CI: 78%-85%) and 83% (77%-87%) among patients who were PF at 24 and 60 months, respectively. For R-single, the negative impact was limited to progression occurring during the first two years following treatment initiation (5-year OS conditional on POD and PF at 12, 24, and 60 months were 67%, 73%, 78%, and 84%, 85%, 82%). In the analysis of POD24, early progression was confirmed to be predictive of a worse OS compared to patients who were PF at 24 months, especially among patients treated with R-chemo (Figure 5).

## DISCUSSION

Among FL patients treated with first-line immunochemotherapy, a progression following primary treatment affected OS negatively. Even though the majority of POD patients progressed within 24 months, it is important to note that the negative impact of POD on OS remained when the progression occurred up to 5 years after treatment initiation. Yet, for patients treated with R-single, POD had less impact on survival and was limited to progression within 2 years of primary treatment. Taken together, these findings are highly clinically relevant, as they imply that timing of progression following standard immunochemotherapy regimens needs to be considered not only before but also beyond the 24-month time point.

To the best of our knowledge, this is the first population-based study of POD and its timing in relation to first-line treatment and survival. Most previous studies have focused on POD24, and have been performed on selected patients included in clinical trials, with lower median age and more favorable baseline characteristics than in our population-based study.^15,16,22,23,25^ The proportion POD in our study was 44% over a median follow-up of 6.1 years, whereas the proportion of POD24 was 28%, in line with previous reports.^15,19^ The proportion R-single treated patients who progressed was high (60%) compared to previous studies,^16,23,24^ which may reflect our population-based setting where high-risk patients are more often selected for R-single. In contrast to other studies that have shown a lower proportion of POD24 after treatment with BR,^18^ we observe a similar proportion of POD (and POD24) in R-CHOP and BR-treated patients. Regardless of first-line treatment strategy, POD was associated with poor prognosis on both the relative and absolute survival scale. While patients treated with R-single had higher proportion of POD, its impact on survival was less dramatic compared to immunochemotherapy-treated patients who experienced POD. This is in line with previous findings on POD24 in patients treated with immunotherapy only^16,17,24^ and may be explained both by more favorable disease, and high efficacy of second-line treatment after initial management with immunotherapy only.

For patients managed with R-single, we show similar OS as those reported for patients treated in clinical trials with immunotherapy alone. For example, Lansigan et al^23^ demonstrated a 5-year OS of 74% among patients treated with immunotherapy who experienced POD24 and 90% among those who did not, compared to 73% and 85% (respectively) in our study. However, our real-world data showed slightly worse survival among patients with POD within 24 months after R-chemo compared to previous studies.^15,17^ In part, this likely also reflects our population-based setting with national guidelines for indolent FL in Sweden recommend R-single also to high-risk patients. Thus, the patients who are selected to receive R-chemo in Sweden may represent an even more pronounced high-risk disease group, which could contribute to the worse survival rates observed. Complementary analyses of cause of death confirmed a strong association between POD and lymphoma-specific death, but also indicated increased mortality due to other causes among immunochemotherapy-treated patients.

The association between POD24 and transformation has varied between studies, with almost 76% of patients who experienced POD24 after BR treatment having transformed disease in one study,^18^ compared with most other studies where approximately 20% of POD24 patients represented transformed disease.^22,24^ In our study, only 62 patients (15%) out of all progressed patients had a morphologically verified transformation, and this did not significantly differ between patients treated with R-CHOP, BR, or R-single. When including also clinically suspected transformation the total percentage was 17%, which is still lower than in previous reports. Thus, the inferior outcome among patients who experience POD after immunochemotherapy in our study is not explained by an increased risk of transformation, as was the case of POD24 in BR-treated patients in British Columbia.^18^

The main strength of this study is the population-based setting with unselected patients in combination with a detailed medical chart review, providing comprehensive data and few missing values. Another strength lies in our choice of statistical approach to analyze the prognostic value of timing of POD. Previous publications have reported POD24, which has laid the ground for research in prognostic markers with its ease of implementation and interpretation. However, POD24 is prone to some issues and limitations. First, the choice of time point can be considered more or less arbitrary, and dichotomizing an exposure implicitly results in loss of information. Moreover, patients progressing even shortly after the 24-month mark will contribute as PF, and patients without progression who die before 24 months will not enter the analysis, resulting in a loss of data. Last, the time scale has a different meaning for PF and progressed, as all patients who are PF at 24 months start their follow-up then, whereas progressed patients may start follow-up shortly after diagnosis. We believe that the prognostic impact of POD is even better studied when progression at different time points are accounted for, all patients are included, and the time scale has the same meaning for all. Regarding limitations, as is inevitable in observational studies, our study comes with an inherent bias due to selection to treatment. Although we have adjusted for several baseline characteristics affecting treatment selection the possibility of residual confounding remains.

The recently reported use of response-adapted therapy in FL^32^ as well as the rapid development of novel agents such as CAR-T cell therapy and bispecific antibodies in FL,^33,34^ augment the need for improved individual risk assessment and treatment choice. The here reported findings on how the impact of progression on OS varies by timing of POD and by treatment constitutes important factors for this assessment. In future research, it is crucial to identify prognostic and predictive markers, not only for patients with POD24 but also for patients progressing after this time point.

## CONCLUSIONS

POD up to five years after first-line systemic treatment has strong prognostic value for patients treated with immunochemotherapy, but less so for patients treated with R-single. In addition to the important finding of POD24 as a marker of subsequent poor survival in previous landmark studies, we should keep in mind that the strength of the association between POD and survival changes continuously over time both before and beyond the 24-month time point. This insight will allow us to improve the clinical prognostic value of POD to support decisions on second-line treatments and inclusion in trials of new therapies. Future studies should aim to determine factors that predict progression at different time points and evaluate whether risk of progression can be reduced through alternative initial management.

## DATA AVAILABILITY STATEMENT

The data underlying this study are available at the National Board of Health and Welfare, Sweden, and Statistics Sweden for investigators with the appropriate approvals, but restrictions apply. However, data can be made available from the authors upon reasonable request for meta-analyses, and with the appropriate approvals of the Swedish Ethical Review Authority (https://etikprovningsmyndigheten.se).

## AUTHOR CONTRIBUTIONS

CEW, TW, and KES conceptualized and designed the study. CEW, SE, BEW, EK, and KES did the data collection. CEW and MJC performed all statistical analyses. CEW, TW, and KES drafted the article. All authors took an active part in interpretation of the results and revisions of the article. All authors have approved the final version to be published.

## DISCLOSURES

The authors have no conflicts of interest to disclose

## SOURCES OF FUNDING

This study/project was funded through grants from the public-private research partnership for real-world data analysis between Karolinska Institutet and Janssen Pharmaceutica NV (Ref: 5-63/2015).

## Supplementary Material



## References

[1] Casulo C. How I manage patients with follicular lymphoma. Br J Haematol. 2019;186:513–523. 31173345 10.1111/bjh.16011

[2] Lo AC Campbell BA Pickles T. Long-term outcomes for patients with limited-stage follicular lymphoma: update of a population-based study. Blood. 2020;136:1006–1010. 32321165 10.1182/blood.2019004588

[3] Kahl BS Yang DT. Follicular lymphoma: evolving therapeutic strategies. Blood. 2016;127:2055–2063. 31659365 10.1182/blood-2016-06-721902

[4] Ardeshna KM Qian W Smith P. Rituximab versus a watch-and-wait approach in patients with advanced-stage, asymptomatic, non-bulky follicular lymphoma: an open-label randomised phase 3 trial. Lancet Oncol. 2014;15:424–435. 24602760 10.1016/S1470-2045(14)70027-0

[5] Tan D Horning SJ Hoppe RT. Improvements in observed and relative survival in follicular grade 1-2 lymphoma during 4 decades: the Stanford University experience. Blood. 2013;122:981–987. 23777769 10.1182/blood-2013-03-491514 PMC3739040

[6] Salles G Mounier N de Guibert S. Rituximab combined with chemotherapy and interferon in follicular lymphoma patients: results of the GELA-GOELAMS FL2000 study. Blood. 2008;112:4824–4831. 18799723 10.1182/blood-2008-04-153189

[7] Witzig TE Vukov AM Habermann TM. Rituximab therapy for patients with newly diagnosed, advanced-stage, follicular grade I non-Hodgkin’s lymphoma: a phase II trial in the North Central Cancer Treatment Group. J Clin Oncol. 2005;23:1103–1108. 15657404 10.1200/JCO.2005.12.052

[8] Junlén HR Peterson S Kimby E. Follicular lymphoma in Sweden: nationwide improved survival in the rituximab era, particularly in elderly women: a Swedish Lymphoma Registry study. Leukemia. 2015;29:668–676. 25151959 10.1038/leu.2014.251

[9] Dinnessen MAW van der Poel MWM Tonino SH. Stage-specific trends in primary therapy and survival in follicular lymphoma: a nationwide population-based analysis in the Netherlands, 1989-2016. Leukemia. 2021;35:1683–1695. 33046819 10.1038/s41375-020-01048-6

[10] Fischer T Zing NPC Chiattone CS. Transformed follicular lymphoma. Ann Hematol. 2018;97:17–29. 29043381 10.1007/s00277-017-3151-2

[11] Solal-Céligny P Roy P Colombat P. Follicular lymphoma international prognostic index. Blood. 2004;104:1258–1265. 15126323 10.1182/blood-2003-12-4434

[12] Federico M Bellei M Marcheselli L. Follicular lymphoma international prognostic index 2: a new prognostic index for follicular lymphoma developed by the international follicular lymphoma prognostic factor project. J Clin Oncol. 2009;27:4555–4562. 19652063 10.1200/JCO.2008.21.3991

[13] Pastore A Jurinovic V Kridel R. Integration of gene mutations in risk prognostication for patients receiving first-line immunochemotherapy for follicular lymphoma: a retrospective analysis of a prospective clinical trial and validation in a population-based registry. Lancet Oncol. 2015;16:1111–1122. 26256760 10.1016/S1470-2045(15)00169-2

[14] Mir F Mattiello F Grigg A. Follicular Lymphoma Evaluation Index (FLEX): a new clinical prognostic model that is superior to existing risk scores for predicting progression-free survival and early treatment failure after frontline immunochemotherapy. Am J Hematol. 2020;95:1503–1510. 32815559 10.1002/ajh.25973 PMC7756469

[15] Casulo C Byrtek M Dawson KL. Early relapse of follicular lymphoma after rituximab plus cyclophosphamide, doxorubicin, vincristine, and prednisone defines patients at high risk for death: an analysis from the National LymphoCare Study. J Clin Oncol. 2015;33:2516–2522. 26124482 10.1200/JCO.2014.59.7534 PMC4879714

[16] Moccia AA Schär S Hayoz S. Prognostic value of POD24 validation in follicular lymphoma patients initially treated with chemotherapy-free regimens in a pooled analysis of three randomized trials of the Swiss Group for Clinical Cancer Research (SAKK). Br J Haematol. 2021;192:1031–1034. 32805081 10.1111/bjh.17045

[17] Sortais C Lok A Tessoulin B. Progression of disease within 2 years (POD24) is a clinically relevant endpoint to identify high-risk follicular lymphoma patients in real life. Ann Hematol. 2020;99:1595–1604. 32417940 10.1007/s00277-020-04025-2

[18] Freeman CL Kridel R Moccia AA. Early progression after bendamustine-rituximab is associated with high risk of transformation in advanced stage follicular lymphoma. Blood. 2019;134:761–764. 31300404 10.1182/blood.2019000258

[19] Morrison VA Shou Y Bell JA. Treatment patterns and survival outcomes in patients with follicular lymphoma: a 2007 to 2015 Humedica Database Study. Clin Lymphoma Myeloma Leuk. 2019;19:e172–e183. 30691994 10.1016/j.clml.2018.12.017

[20] Provencio M Royuela A Torrente M. Prognostic value of event-free survival at 12 and 24 months and long-term mortality for non-Hodgkin follicular lymphoma patients: a study report from the Spanish Lymphoma Oncology Group. Cancer. 2017;123:3709–3716. 28608996 10.1002/cncr.30795

[21] Evens AM Hong F Habermann TM. A three-arm randomized phase II study of bendamustine/rituximab with bortezomib induction or lenalidomide continuation in untreated follicular lymphoma: ECOG-ACRIN E2408. Clin Cancer Res. 2020;26:4468–4477. 32532790 10.1158/1078-0432.CCR-20-1345 PMC7722783

[22] Seymour JF Marcus R Davies A. Association of early disease progression and very poor survival in the GALLIUM study in follicular lymphoma: benefit of obinutuzumab in reducing the rate of early progression. Haematologica. 2019;104:1202–1208. 30573503 10.3324/haematol.2018.209015 PMC6545851

[23] Lansigan F Barak I Pitcher B. The prognostic significance of PFS24 in follicular lymphoma following firstline immunotherapy: a combined analysis of 3 CALGB trials. Cancer Med. 2019;8:165–173. 30575311 10.1002/cam4.1918 PMC6346218

[24] Lockmer S Østenstad B Hagberg H. Chemotherapy-free initial treatment of advanced indolent lymphoma has durable effect with low toxicity: results from two nordic lymphoma group trials with more than 10 years of follow-up. J Clin Oncol. 2018;36:3315–3323. 10.1200/JCO.18.00262 30285560

[25] Casulo C Dixon JG Le-Rademacher J. Validation of POD24 as a robust early clinical end point of poor survival in FL from 5225 patients on 13 clinical trials. Blood. 2022;139:1684–1693. 34614146 10.1182/blood.2020010263 PMC9974165

[26] Yoon SE Cho J Kim WS. Impact of transformation on the survival of patients diagnosed with follicular lymphoma that progressed within 24 months. J Cancer. 2021;12:2488–2497. 33854610 10.7150/jca.54434 PMC8040724

[27] The Swedish Lymphoma Group. Annual Report From The National Quality-of-care Register For Lymphoma, diagnosis period 2000-2020. [In Swedish] 2021. https://cancercentrum.se/globalassets/cancerdiagnoser/blod-lymfom-myelom/lymfom/rapporter/lymfom-arsrapport-2000-2020.pdf. Accessed February 20, 2023.

[28] Crowther MJ Lambert PC. Parametric multistate survival models: flexible modelling allowing transition-specific distributions with application to estimating clinically useful measures of effect differences. Stat Med. 2017;36:4719–4742. 28872690 10.1002/sim.7448

[29] Rajamäki A Sunela K Prusila REI. Female patients with follicular lymphoma have a better prognosis if primary remission lasts over 24 months. Leuk Lymphoma. 2021;62:1639–1647. 33546574 10.1080/10428194.2021.1872073

[30] Batlevi CL Sha F Alperovich A. Positron-emission tomography-based staging reduces the prognostic impact of early disease progression in patients with follicular lymphoma. Eur J Cancer. 2020;126:78–90. 31927165 10.1016/j.ejca.2019.12.006 PMC7331469

[31] Bitansky G Avigdor A Vasilev E. Progression of disease within 24 months of initial therapy (POD24) detected incidentally in imaging does not necessarily indicate worse outcome. Leuk Lymphoma. 2020;61:2645–2651. 32643497 10.1080/10428194.2020.1786554

[32] Luminari S Manni M Galimberti S. Response-adapted postinduction strategy in patients with advanced-stage follicular lymphoma: the FOLL12 Study. J Clin Oncol. 2022;40:729–739. 34709880 10.1200/JCO.21.01234

[33] Fowler NH Dickinson M Dreyling M. Tisagenlecleucel in adult relapsed or refractory follicular lymphoma: the phase 2 ELARA trial. Nat Med. 2021;28:325–332. 34921238 10.1038/s41591-021-01622-0

[34] Budde LE Sehn LH Matasar MJ. Mosunetuzumab monotherapy is an effective and well-tolerated treatment option for patients with Relapsed/Refractory (R/R) Follicular Lymphoma (FL) who have received ≥2 prior lines of therapy: pivotal results from a phase I/II study. Blood. 2021;138(Suppl 1):127–127.

