# Supplementary material for: Survival by First-line Treatment Type and Timing of Progression Among Follicular Lymphoma Patients: A National Population-based Study in Sweden
Source: Hemasphere. 2023 Feb 23;7(3):e838. doi: 10.1097/HS9.0000000000000838 (PMC9953041; doi:10.1097/HS9.0000000000000838)
Supplement: Supplementary file 1 [file hs9-7-e838-s001.docx]

**SUPPLEMENTARY TABLE 1** Hazard ratios (HRs) with 95% confidence intervals (CIs) comparing all-cause mortality by patient and clinical characteristics. Estimated from univariable Cox proportional hazards models.

|  | **Variable** | **Total** | **HR (95% CI)** |
| --- | --- | --- | --- |
| **Year of diagnosis, n (%)** | 2007-2010 | 471 (49.7) | 1.00 |
|  | 2011-2014 | 477 (50.3) | 0.99 (0.78-1.27) |
| **Age at diagnosis, n (%)** | ≤49 | 123 (13.0) | 1.00 |
|  | 50-64 | 290 (30.6) | 2.16 (1.19-3.93) |
|  | 65-74 | 316 (33.3) | 3.53 (1.98-6.31) |
|  | ≥75 | 219 (23.1) | 8.18 (4.60-14.5) |
| **Sex, n (%)** | Male | 482 (50.8) | 1.00 |
|  | Female | 466 (49.2) | 0.77 (0.61-0.98) |
| **Stage, n (%)** | Ann-Arbor II | 187 (19.7) | 1.00 |
|  | Ann-Arbor III | 326 (34.4) | 0.89 (0.64-1.24) |
|  | Ann-Arbor IV | 429 (45.3) | 1.06 (0.78-1.43) |
|  | Missing | 6 (0.6) | . |
| **FLIPI, n (%)** | Low risk | 185 (19.5) | 1.00 |
|  | Intermediate risk | 294 (31.0) | 1.44 (0.98-2.13) |
|  | High risk | 455 (48.0) | 2.05 (1.43-2.93) |
|  | Missing | 14 (1.5) | . |
| **Performance status, n (%)** | 0 | 645 (68.0) | 1.00 |
|  | 1 | 225 (23.7) | 2.35 (1.83-3.03) |
|  | 2+ | 60 (6.3) | 4.29 (2.80-6.56) |
|  | Unclear | 18 (1.9) | 2.21 (1.08-4.51) |
|  | **Grade, n (%)** |  |  |
|  | 1 | 222 (23.4) | 1.00 |
|  | 2 | 408 (43.0) | 0.93 (0.69-1.25) |
|  | 3A | 184 (19.4) | 0.94 (0.65-1.35) |
|  | Low grade UNS | 27 (2.9) | 0.51 (0.21-1.27) |
|  | Unclear | 107 (11.3) | 1.50 (1.02-2.19) |

**SUPPLEMENTARY TABLE 2** Unadjusted and adjusted hazard ratios (HRs) with 95% confidence intervals (CIs) comparing lymphoma-specific and other-cause mortality between patients with progression of disease (POD) and patients still progression-free (PF), by first-line treatment. Among 48 (13%) of deceased patients the cause of death was not known, and hence these patients did not contribute to the analyses.

|  | **Lymphoma-specific mortality**  (events=168) | | **Other-cause mortality**  (events=140) | |
| --- | --- | --- | --- | --- |
|  | **HR* (95% CI)** | **HR** (95% CI)** | **HR* (95% CI)** | **HR** (95% CI)** |
| **R-chemo** |  |  |  |  |
| PF | 1.00 | 1.00 | 1.00 | 1.00 |
| POD | 42.5 (24.2-74.7) | 41.9 (23.9-73.6) | 1.86 (1.12-3.07) | 2.39 (1.44-3.97) |
| **R-single** |  |  |  |  |
| PF | 1.00 | 1.00 | 1.00 | 1.00 |
| POD | 39.1 (11.8-129.4) | 36.1 (10.9-119.3) | 1.35 (0.68-2.69) | 1.35 (0.67-2.70) |

** Estimates from Cox proportional hazards models adjusted for time since 1^st^ line treatment.*

*** Estimates from Cox proportional hazards models additionally adjusted for age at diagnosis (categorised), sex, calendar year of diagnosis (categorised), and FLIPI.*

**SUPPLEMENTARY FIGURE 1.** Illustration of illness-death model used to estimate transition rates between the illustrated states, used for the prediction of survival probabilities.

**ALIVE and PF**

start = 948

end = 384

**DEAD before POD**

start = 0

end = 150

**DEAD after POD**

start = 0

end = 232

n = 414

**POD**

start = 0

end = 208

n = 150

n = 206
